# Supplementary material for: Content-rich biological network constructed by mining PubMed abstracts
Source: BMC Bioinformatics. 2004 Oct 8;5:147. doi: 10.1186/1471-2105-5-147 (PMC528731; doi:10.1186/1471-2105-5-147)
Supplement: Additional File 5 — The original Chilibot query results of the term "long-term potentiation (LTP)" and 22 other terms, limiting the latest references analyzed to the years 1990, 1995, 2000, and 2004. [file 1471-2105-5-147-S5.bz2 › chilibotAdditionalFile5/ltp1995/html/ZIF268_ACTIN.html]

 


 **ZIF268** and **ACTIN** 
  
Found 3 abstracts in PubMed,  **3 abstracts were retrieved and analyzed**.  


---

 Search Google  |
 PDF files only 
|  EDU domain only 

---

**Interactive relationship** (e.g. stimulation, inhibition, etc)

- The 5 flanking region of  **zif268**  contains sequences that resemble known regulatory elements, including four CC A or T 6GG sequences similar to the core serum response elements SREs found upstream of c fos and  **actin**  genes.  Ref: 2513479 Mol Cell Biol, 1989

**Parallel relationship** (e.g. studied together, co-existance, homology, etc.)

- Band 3 also formed with gamma  **actin**  and  **zif268**  3 SRE probes.  Ref: 8063733 J Biol Chem, 1994
